# Investigating the effectiveness of adjuvant therapy for patients with hormone receptor-positive ductal carcinoma in situ

**DOI:** 10.1371/journal.pone.0262934

**Published:** 2022-01-28

**Authors:** Chi-Jui Tsai, Ho-Yin Huang, Fang-Ming Chen, Yi-Hsin Yang, Li-Chia Chen, Kun-Pin Hsieh

**Affiliations:** 1 School of Pharmacy, College of Pharmacy, Kaohsiung Medical University, Kaohsiung, Taiwan; 2 Department of Pharmacy, Kaohsiung Medical University Hospital, Kaohsiung Medical University, Kaohsiung, Taiwan; 3 Division of Breast Surgery, Department of Surgery, Kaohsiung Medical University Hospital, Kaohsiung, Taiwan; 4 Department of Surgery, Kaohsiung Municipal Ta-Tung Hospital, Kaohsiung, Taiwan; 5 Department of Surgery, Faculty of Medicine, College of Medicine, Kaohsiung Medical University, Kaohsiung, Taiwan; 6 National Institute of Cancer Research, National Health Research Institutes, Tainan, Taiwan; 7 Centre for Pharmacoepidemiology and Drug Safety, Division of Pharmacy and Optometry, School of Health Sciences, Faculty of Biology, Medicine and Health, University of Manchester, Manchester Academic Health Science Centre, Manchester, Lancashire, United Kingdom; Centro per lo Studio e la Prevenzione Oncologica, ITALY

## Abstract

**Background:**

This study compared the recurrence risk of single versus dual adjuvant radiotherapy (RT) and hormonal therapy (HT) following breast-conserving surgery (BCS) in patients with hormone receptor-positive ductal carcinoma in situ (DCIS).

**Methods:**

This retrospective cohort study used the Taiwan Cancer Registry database linking to the Taiwan National Health Insurance data from 2011 to 2016. We compared the recurrence risk between BCS-based regimens in Cox regressions and presented as adjusted hazard ratio (HR) and 95% confidence interval (95%CI).

**Results:**

The 1,836 study cohort with a low-to-intermediate risk of recurrence was grouped into BCS alone (6.1%), BCS+RT (6.2%), BCS+HT (23.4%) and BCS+HT+RT (64.3%) according to the initial treatments. During the follow-up (median: 3.3 years), the highest 5-year recurrence-free survival rate was in BCS+RT (94.1%) group and followed by BCS+HT+RT (92.8%), BCS+HT (87.4%) and BCS alone (84.9%). Of the single adjuvant therapies, RT was more effective than HT. Both BCS+HT (HR: 1.52, 95%CI: 0.99–2.35) and BCS+RT (HR: 1.10, 95%CI: 0.50–2.41) did not significantly increase recurrence risk comparing against the BCS+HT+RT group.

**Conclusion:**

Single adjuvant demonstrated a similar subsequent recurrence risk with dual adjuvant. This study supports the proposition to de-escalate adjuvant treatments in patients with low-to-intermediate risk of DCIS recurrence.

## Introduction

Ductal carcinoma in situ (DCIS) is non-invasive and typically curable breast cancer that accounts for approximately 20%–25% of all newly diagnosed breast cancer in the United States and 17%–34% of mammography-detected cases [1–3]. According to the National Comprehensive Cancer Network (NCCN) guideline, treatment options for DCIS include mastectomy alone or breast-conserving surgery (BCS) with whole breast radiation therapy (RT) (with or without radiation boost) and with or without hormone therapy (HT) [4].

Previous clinical trials have shown that both adjuvant RT and HT decrease the reduced the ipsilateral breast tumor recurrence (IBTR) (i.e., either recurrent DCIS or invasive cancer) rate by approximately 50% and 30%, respectively [5–7]. A meta-analysis on four randomized trials found that compared with BCS alone, the adjuvant RT after BCS significantly decreased 15.2% of the ten-year absolute risk of any IBTR (BCS+RT vs. BCS: 12.9% vs. 28.1%; P<0.00001), despite no significant effect on breast cancer mortality or all-cause mortality [8]. Moreover, a case-series analysis at a single-center in South Korea found that adjuvant HT combining RT with BCS further reduced LR by 30% in 50 women with hormone receptor (HR)-positive (+) DCIS, which is regarded with a low risk of recurrence [9, 10].

Although single (RT only) or dual (RT combining HT) adjuvant therapies have been demonstrated to reduce the LR of DCIS significantly, no significant difference was found in the mortality caused by invasive breast cancer recurrence. Therefore, nowadays, increasing attention has been raised on the de-escalation of dual adjuvant RT and HT following BCS [11] when considering the benefit of controlling local recurrence and the undesirable adverse effects, e.g., RT associated cardiovascular disease and rare malignancies [12, 13] and HT associated thromboembolic events [14]. Besides, adjuvant HT is recommended using consecutively up to five years post-surgery for women with HR-positive DCIS. Adherence to HT was generally suboptimal due to side effects and consequently reduced the treatment effectiveness [15–19]. However, these long-term adverse consequences were generally not measured in randomized controlled trials.

Several risk predictive tools have been developed to classify the risk of recurrence into three categories of high, intermediate and low risk based on either the clinicopathological factors (e.g., Van/Nuys prognostic index and system established by Smith *et al*.) [20, 21] or incorporating genetic factors (e.g., Oncotype DX®) [22]. By incorporating these risk scoring systems and patients’ bio-molecular profiles, there may be a great potential to inform clinical decision-making on de-escalating adjuvant therapies in patients with (HR)-positive (+) DCIS. Nevertheless, these risk scoring tools are needed to be validated in a larger population covering a wide range of ethnicities and socio-diversity. Therefore, this study aimed to investigate the clinical effectiveness of single (RT or HT) and dual (RT and CT) adjuvant therapy following BCS in patients with a low-to-intermediate recurrence risk of DCIS using population-based real-world data. The objectives were to investigate patients’ treatment patterns, compare the effectiveness between different treatment options and investigate factors associated with the risk of recurrence.

## Materials and methods

### Study design and data sources

This retrospective cohort study used population-level claim-based data from the National Health Insurance (NHI) database, Taiwan Cancer Registry (TCR), and the Breast Cancer Screening Database from 2010 to 2017. In 1995, Taiwan implemented a single-payer NHI system to enhance medical care coverage, which reached over 99.9% of all population in 2014 [23]. The NHI database includes comprehensive claims data for reimbursements on ambulatory, inpatient, emergency, and Chinese medicine visits. The TCR provided archives information on cancer diagnosis, and records additional supplementary information. In 2015, biennial breast cancer screening programs were extended to women aged 45–69 years [24]; accordingly, the Breast Cancer Screening Database provided associated information, such as mammographic results, family history, and menstruation status. The study period was selected for the completion of biomarker information in TCR. This study protocol was approved by the Institutional Review Board of Kaohsiung Medical University Hospital (KMUHIRB-EXEMPT(I)-20180037).

### Cohort selection

The study population was adult women (age ≥ 20 years) with newly-diagnosed HR(+) (i.e., estrogen receptor-positive and or progesterone receptor-positive) DCIS and no other concomitant cancers who had undergone BCS as the initial treatment. Patients with DCIS were identified by screening the TCR from 2011 to 2016 for the DCIS-related ICD-O-3 Topography codes C50.x and Morphology codes (8201, 8230, 8500, 8501, 8503, 8507, and 8522). The date of the first DCIS diagnosis was defined as the index date. Patients with ICD-9 codes for other cancers (i.e., 140–208, 230–239) recorded before the index date, HR(−) breast cancer and not undergone BCS as the initial breast cancer management were excluded.

**Fig 1:**
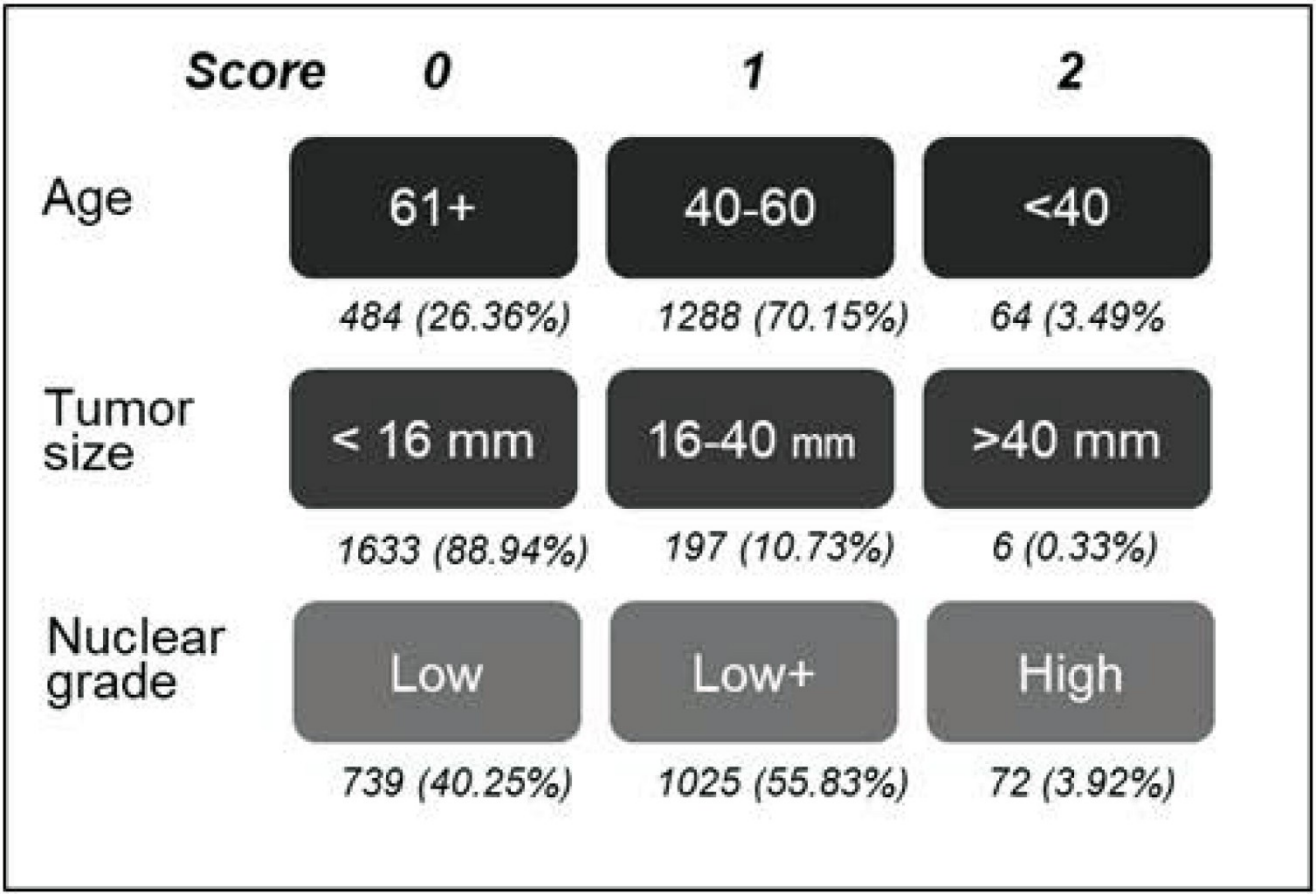
Scoring system for the risk of recurrence on the study cohort. According to Smith *et al*. [21], the total score (from the age, tumor size and nuclear grade) 0–3 is categorized as low-to-intermediate risk, 4–6 is regarded as high risk.

Furthermore, we adopted the scoring system proposed by Smith *et al*. (2006) to stratify the risk of recurrence in the study cohort [21]. A cumulative score was derived from each patient’s age at diagnosis, tumor size, and nuclear grade (Fig 1). Those who had a total score of >3 points were excluded to ensure that the study cohort was at low-to-intermediate risk of recurrence. Excluded patients (i.e., with a high risk of recurrence) were obtained in an additional analysis (S1 Fig). Moreover, as previous studies indicated the premenopausal status as a prognostic factor for DCIS recurrence [25–27], in a sub-analysis, we grouped the study cohort according to the menopausal status [18, 28]. If the information on menstruation status was missing, the age of diagnosis older than 52 years, the median menopausal age in Taiwan [29] was used as a proxy to classify the menopausal status.

### Exposure

The initial breast cancer treatments were identified within six months after the DCIS was diagnosed. According to the patients ’ initial breast cancer treatments, the study cohort was then grouped into four BCS-based regimens, including BCS alone, BCS+RT, BCS+HT and BCS+HT+RT. Corresponding procedure codes of BCS and RT and prescription codes of HT were used to screen the claims. HT included tamoxifen (ATC code L02BA01), anastrozole (ATC code L02BG03), letrozole (ATC code L02BG04), and exemestane (ATC code L02BG06).

### Outcome measure

The study cohort was followed from the index date to the recurrence event, death, or end of the study period (December 31, 2017), whichever occurred first. The primary outcome, any recurrent events (i.e., either recurrent DCIS or invasive cancer), as defined by a proxy of resuming treatment, i.e., any surgery or chemotherapy records identified in the NHI database after nine months from the index date because the recurrence was not recorded in the claim-based data. The completion of initial breast cancer treatments for DCIS was assumed to be up to nine months following the index diagnosis date. Based on clinical specialists’ experience, the completion of initial breast cancer management typically requires up to 6 months in clinical practice [30]. Moreover, as DCIS is a relatively benign condition, treatment initiation may be delayed for up to three months [31]. The time to LR was also measured to calculate the recurrence-free survival (RFS) rate over time.

### Covariates

Patients’ demographic (age at diagnosis, year diagnosed), socioeconomic (residential areas of NHI divisions, income rank of NHI registration), lifestyle (obesity, or body mass index of >25, smoking, and alcohol consumption) factors, comorbidity index, medical history (menstruation status, mammography, family history of breast cancer) and tumor features (size, nuclear grade, and histology type) were retrieved. Patients were categorized into three age groups, i.e. 20–50, 51–70, and >70 years. The insurance income ranking was used as a proxy for the monthly income level. Patients’ comorbidities were identified within the preceding year of the index date to calculate the Charlson’s comorbidity index (CCI) score [32] and further categorized into four groups (i.e. 0, 1, 2, and ≥3 points of CCI). Data on mammography results, family history, and menstruation status before the index date were extracted from the Breast Cancer Screening Database.

### Analysis

The RFS rate was presented in a Kaplan–Meier survival curve and compared the four BCS-based regimens using the log-rank test. The Cox proportional hazards model was used to analyze the LR risk comparing BCS, BSC+HT, BCS+RT against BCS+RT+HT and adjust for covariates, including residential areas, diagnosed a year, and insurance income rank in the multivariable analysis. The results were adjusted hazard ratios (HRs) and 95% confidence intervals (95%CIs).

Moreover, a sub-analysis was conducted to assess the recurrence rates comparing treatment regimens with or without HT (i.e. BCS±RT+HT vs. BCS±RT) in patients at the pre and post-menopausal status [18, 28]. Furthermore, an additional analysis of the RFS rate was conducted in the groups of patients with a high-risk of recurrence. The Kaplan–Meier survival curve and the log-rank test comparing among the four different BCS-based regimens were presented in S1 Fig.

Data management, computation, and analysis were performed using the SAS software (version 9.4: SAS Institutes, Inc., Cary, NC, US).

## Results

### Characteristics of the study cohort

During the 6-year inclusion period (2011–2016), 2,911 patients with HR(+) DCIS received BCS as the initial breast cancer management. Of them, 1836 were identified as the low-intermediate-risk cohort and included in the analysis. The median follow-up period was 3.3 years; the mean age (± standard deviation) at diagnosis was 53.6±9.7 years, and 51.4% of the patients were postmenopausal women. The mean tumor size was 9.3±6.9 mm; most patients (86.1%) exhibited a non-comedo-type lump. Most patients had a CCI score of 0 (60.4%), were not obese (72.0%), did not smoke (87.9%), casually drink or did not drink (88.1%) (Table 1). All patients were at low-intermediate-risk of recurrence (scored 1–3 according to Smith *et al*. (2006) [21]) and a high proportion of patients aged 40–60 years (70.2%) had <16-mm tumor size (88.9%) and low (40.3%) to intermediate (55.8%) nuclear grade (Fig 1).

**Table 1:** Baseline characteristics of the study cohort.

	Total (N = 1836)	BCS alone (n = 111, 6.1%)	BCS+HT (n = 430, 23.4%)	BCS+RT (n = 114, 6.2%)	BCS+HT+RT (n = 1181, 64.3%)	p value
***Follow-up period (Years)***						
Mean ±SD	3.5 ±1.7	3.5 ±1.8	3.3 ±1.7	3.8 ±1.7	3.5 ±1.7	0.0254
Median (Q1, Q3)^†^	3.3 (2.1–4.8)	3.3 (1.9–5.0)	3.0 (1.9–4.6)	3.7 (2.5–5.2)	3.4 (2.2–4.9)	
***Age of diagnosis***						
Mean ±SD	53.6 ±9.7	52.6 ±11.5	55.0 ±10.8	52.7 ±10.3	53.3 ±8.9	0.0053
Median (Q1, Q3)^†^	52.0 (47–61)	51.0 (45–61)	54.0 (47–63)	52.0 (46–61)	52.0 (47–60)	
***Age ranks (%)***						
20–50 y/o	785 (42.8)	55 (49.6)	168 (39.1)	52 (45.6)	510 (43.2)	<0.0001
51–70 y/o	979 (53.3)	47 (42.3)	231 (53.7)	57 (50.0)	644 (54.5)	
>70 y/o	72 (3.9)	9 (8.1)	31 (7.2)	5 (4.4)	27 (2.3)	
***Diagnosed year (%)***						
2011	212 (11.6)	14 (12.6)	48 (11.2)	19 (16.7)	131 (11.1)	0.0255
2012	249 (13.6)	21 (18.9)	41 (9.5)	13 (11.4)	174 (14.7)	
2013	290 (15.8)	18 (16.2)	70 (16.3)	19 (16.7)	183 (15.5)	
2014	328 (17.9)	19 (17.1)	68 (15.8)	26 (22.8)	215 (18.2)	
2015	387 (21.1)	15 (13.5)	101 (23.5)	25 (21.9)	246 (20.8)	
2016	370 (20.2)	24 (21.6)	102 (23.7)	12 (10.5)	232 (19.6)	
***Characteristics of tumor***						
Tumor size (mm)						
Mean ±SD	9.3 ±6.9	7.5 ±5.1	9.0 ±7.9	10.4 ±7.5	9.5 ±6.6	0.0053
Grade (%)						
Low	739 (40.3)	63 (56.8)	195 (45.4)	37 (32.5)	444 (37.6)	<0.0001
Intermediate	1025 (55.8)	45 (40.5)	221 (51.4)	65 (57.0)	694 (58.8)	
High	72 (3.9)	3 (2.7)	14 (3.3)	12 (10.5)	43 (3.6)	
Histology (%)						
Comedo type	256 (13.9)	9 (8.1)	43 (10.0)	16 (14.0)	188 (15.9)	0.0057
None-comedo type	1580 (86.1)	102 (91.9)	387 (90.0)	98 (86.0)	993 (84.1)	
***Screening data***						
Family history (%)						
Negative	1119 (61.0)	55 (49.6)	251 (58.4)	70 (61.4)	743 (62.9)	0.0076
Positive	98 (5.3)	4 (3.6)	17 (4.0)	9 (7.9)	68 (5.8)	
Missing	619 (33.7)	52 (46.9)	162 (37.7)	35 (30.7)	370 (31.3)	
Menstruation status (%)						
Premenopausal	892 (48.6)	56 (50.5)	187 (43.5)	59 (51.8)	590 (50.0)	0.1128
Postmenopausal	944 (51.4)	55 (49.6)	243 (56.5)	55 (48.3)	591 (50.0)	
Suspect malignancy (%)						
Negative	375 (20.4)	18 (16.2)	89 (20.7)	21 (18.4)	247 (20.9)	0.0114
Positive	842 (45.9)	41 (36.9)	179 (41.6)	58 (50.9)	564(47.8)	
Missing	619 (33.7)	52 (46.9)	162 (37.7)	35 (30.7)	370 (31.3)	
**CCI (%)**						
0	1109 (60.4)	72 (64.9)	254 (59.1)	66 (57.9)	717 (60.7)	0.8754
1	413 (22.5)	25 (22.5)	101 (23.5)	26 (22.8)	261 (22.1)	
2	188 (10.2)	8 (7.2)	50(11.6)	13 (11.4)	117 (9.9)	
≥3	126 (6.9)	6 (5.4)	25 (5.8)	9 (7.9)	86 (7.3)	
***Lifestyle***						
Obesity (%)^‡^						
Negative	1321 (72.0)	76 (68.5)	314 (73.0)	93 (81.6)	838 (71.0)	0.1523
Positive	345 (18.8)	21 (18.9)	81 (18.8)	11 (9.7)	232 (19.6)	
Missing	170 (9.3)	14 (12.6)	35 (8.1)	10 (8.8)	111 (9.4)	
Smoking (%)						
Negative	1613 (87.9)	92 (82.9)	387 (90.0)	99 (86.8)	1035 (87.6)	0.2046
Positive	223 (12.2)	19 (17.1)	43 (10.0)	15 (13.2)	146 (12.4)	
Drinking (%)^§^						
Negative	1617 (88.1)	88 (79.3)	382 (88.8)	101 (88.6)	1046 (88.6)	0.0333
Positive	219 (11.9)	23 (20.7)	48 (11.2)	13 (11.4)	135 (11.4)	
***Insured profile***						
Insurance income rank (%)^¶^						
< = 22000 NTD	232 (12.6)	16 (14.4)	58 (13.5)	15 (13.2)	143 (12.1)	0.8189
> 22000 NTD	1604 (87.4)	95 (85.6)	372 (86.5)	99 (86.8)	1038 (87.9)	
***Residential areas (%)***						
Northern area	1100 (59.9)	72 (64.9)	238 (55.4)	83 (72.8)	707 (59.9)	0.0012
Central area	254 (13.8)	6 (5.4)	78 (18.1)	10 (8.8)	160 (13.6)	
Southern/Eastern area	482 (26.3)	33 (29.7)	114 (26.5)	21 (18.4)	314 (26.6)	

Abbreviations: BCS, breast-conserving surgery; RT, radiation therapy; HT, hormonal therapy; SD, standard deviation; CCI, Charlson Comorbidity Index; NTD, New Taiwan Dollar;

^†^Q1: the 25^th^ percentile, Q3: the 75^th^ percentile.

^‡^Obesity was defined as a body mass index of >25 according to Health Promotion Administration

^§^Those recorded as never-drink or occasionally drank.

^¶^The income-related insurance payment category set by the Bureau of National Health Insurance in Taiwan; 1 NTD = 0.03 USD in 2019

### Characteristics of cohort receiving different BCS-based regimens

Of the four BCS-based initial treatments, most of the study cohort received BCS+HT+RT (64.3%), followed by BCS+HT (23.4%), BCS+RT (6.2%), and BCS alone (6.1%) (Table 1). Compared with the other treatment groups, the BCS alone group (n = 111) had the highest proportion of patients with low nuclear grade tumors (56.8%) (*P*<0.0001). Moreover, the BCS+RT group reported the largest tumor size of 10.4±7.5 mm (*P* = 0.0053) and the highest nuclear grade (10.5%). Of patients who received only one adjuvant therapy, the BCS+HT group (n = 430) had older diagnosis age, smaller tumor size, a higher proportion of low nuclear grade and non-comedo-type tumors than the BCS+RT group (n = 114).

### Recurrence-free survival rate and associated factors

**Fig 2:**
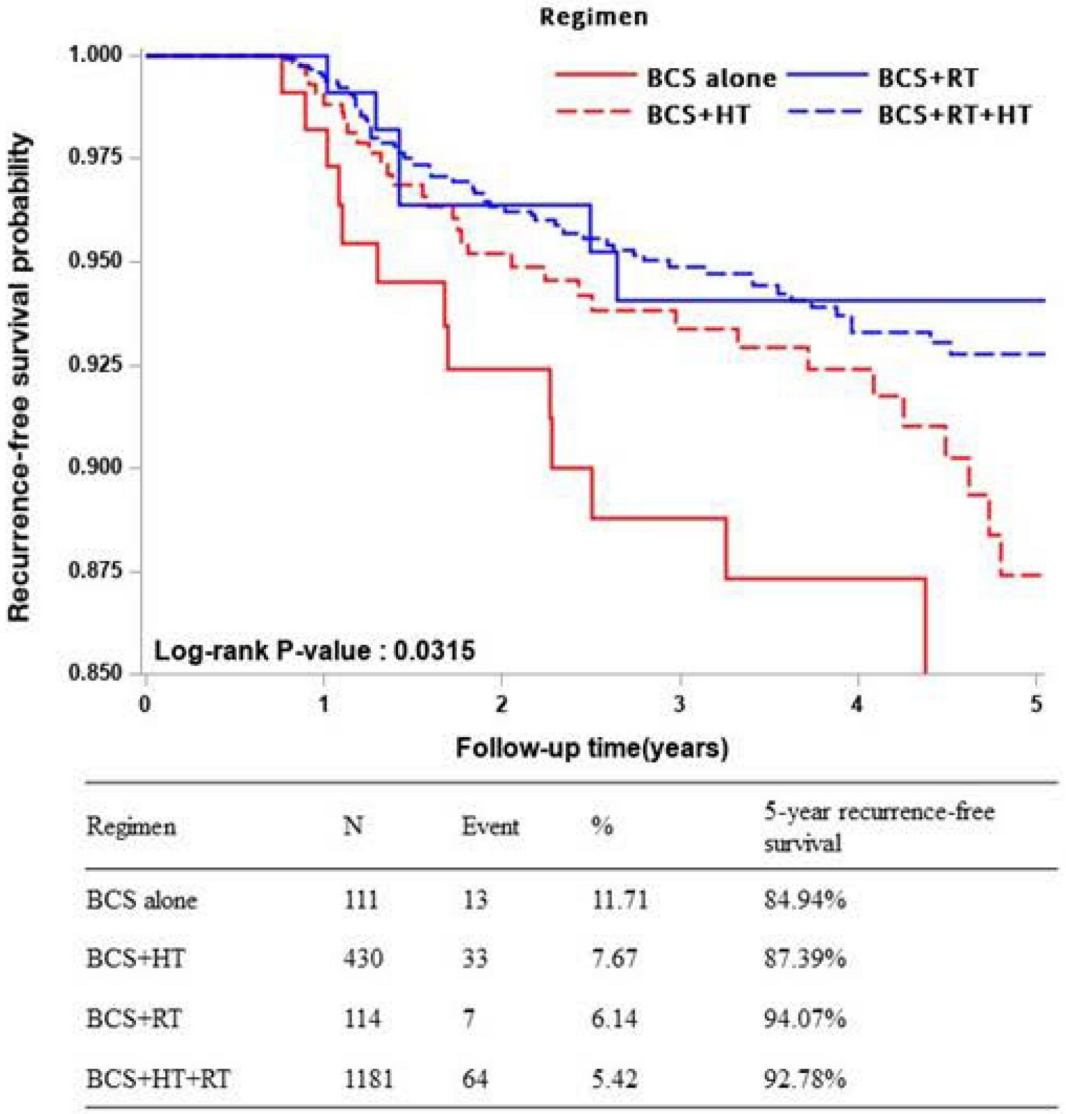
Kaplan Meier plot of cumulative recurrence-free survival among BCS-based regimens. Abbreviations: BCS, breast-conserving surgery; HT, hormone therapy; RT, radiation therapy; RFS, recurrence-free survival.

During the follow-up period, the proportion of patients developed the recurrent was highest in the BCS alone group (11.71%, n = 13), and followed by BCS+HT (7.67%, n = 33), BCS+RT (6.14%, n = 7) and BCS+HT+RT (5.42%, n = 64) groups. Likewise, the BCS alone group had the lowest five-year RFS rate (84.94%) compared with the BCS+HT (87.39%), BCS+RT (94.07%), and BCS+HT+RT (92.78%) groups (log-rank test, *P* = 0.0315) (Fig 2). Consistently, after adjusting all covariates, the BCS alone group showed significantly higher recurrence risk compared with the BCS+HT+RT group (adjusted HR: 2.05, 95%CI: 1.11–3.78, *P* = 0.0216). However, there was no significant difference of recurrent rate comparing BCS+HT (adjusted HR: 1.52, 95%CI: 0.99–2.35, *P* = 0.0582) and BCS+RT (adjusted HR: 1.10, 95%CI: 0.50–2.41, *P* = 0.8186) groups against the BCS+HT+RT group (Table 2).

**Table 2:** Univariate and multivariable adjusted hazard ratios of covariates for breast cancer recurrence.

Characteristic	Univariate HR	P value	Adjusted HR^†^	P value
**BCS-based regimen**				
BCS+HT+RT	1.00		1.00	
BCS alone	2.20 (1.21–3.99)	0.0097	2.05 (1.11–3.78)	0.0216
BCS+HT	1.50 (0.98–2.28)	0.0598	1.52 (0.99–2.35)	0.0582
BCS+RT	1.06 (0.48–2.31)	0.8892	1.10 (0.50–2.41)	0.8186
**Age ranks**				
20–50 y/o	1.00		1.00	
51–70 y/o	0.60 (0.42–0.88)	0.0083	0.73 (0.38–1.39)	0.3335
>70 y/o	0.88 (0.35–2.18)	0.7769	0.73 (0.23–2.30)	0.5889
***Characteristics of tumor***				
Tumor size	1.01 (0.98–1.03)	0.5909	1.01 (0.99–1.04)	0.3177
Nuclear grade				
Low	1.00		1.00	
Intermediate	0.87 (0.61–1.26)	0.4716	1.03 (0.70–1.52)	0.8756
High	0.42 (0.10–1.72)	0.2268	0.60 (0.14–2.55)	0.4843
Histology				
Non-comedo type	1.00		1.00	
Comedo type	1.24 (0.68–2.26)	0.4753	1.19 (0.64–2.22)	0.5906
***Screening data***				
Menopausal status				
Premenopausal	1.00		1.00	
Postmenopausal	0.69 (0.48–0.99)	0.0435	0.91 (0.47–1.75)	0.7801
Previously suspected as malignancy^‡^				
Negative	1.00		1.00	
Positive	1.65 (0.86–3.15)	0.1312	1.75 (0.89–3.45)	0.1072
Family history^‡^				
Negative	1.00		1.00	
Positive	0.33 (0.08–1.34)	0.1216	0.38 (0.09–1.57)	0.1816
CCI				
0	1.00		1.00	
1	0.96 (0.60–1.52)	0.8448	1.14 (0.71–1.83)	0.5938
2	1.31 (0.76–2.26)	0.3261	1.55 (0.89–2.70)	0.1259
≥3	0.99 (0.47–2.05)	0.9697	1.46 (0.68–3.12)	0.3321
***Lifestyle***				
Smoking				
Negative	1.00		1.00	
Positive	1.00 (0.57–1.75)	0.9910	1.20 (0.51–2.85)	0.6741
Alcohol consumption				
Negative	1.00		1.00	
Positive	0.94 (0.53–1.67)	0.8293	0.81 (0.33–1.98)	0.6395
Obesity^‡^^,^^§^				
Negative	1.00		1.00	
Positive	0.67 (0.39–1.14)	0.1378	0.70 (0.40–1.21)	0.2002

Abbreviations: BCS, breast-conserving surgery; HT, hormonal therapy; RT, radiation therapy

^†^The multivariable analysis was adjusted by the covariates, including residential areas, diagnosed a year, and insurance income rank.

^‡^We created another category for the missing data and adjusted them in the regression model.

^§^Obesity was defined as a body mass index of >25 according to the Health Promotion Administration

### Sub-analysis

**Fig 3:**
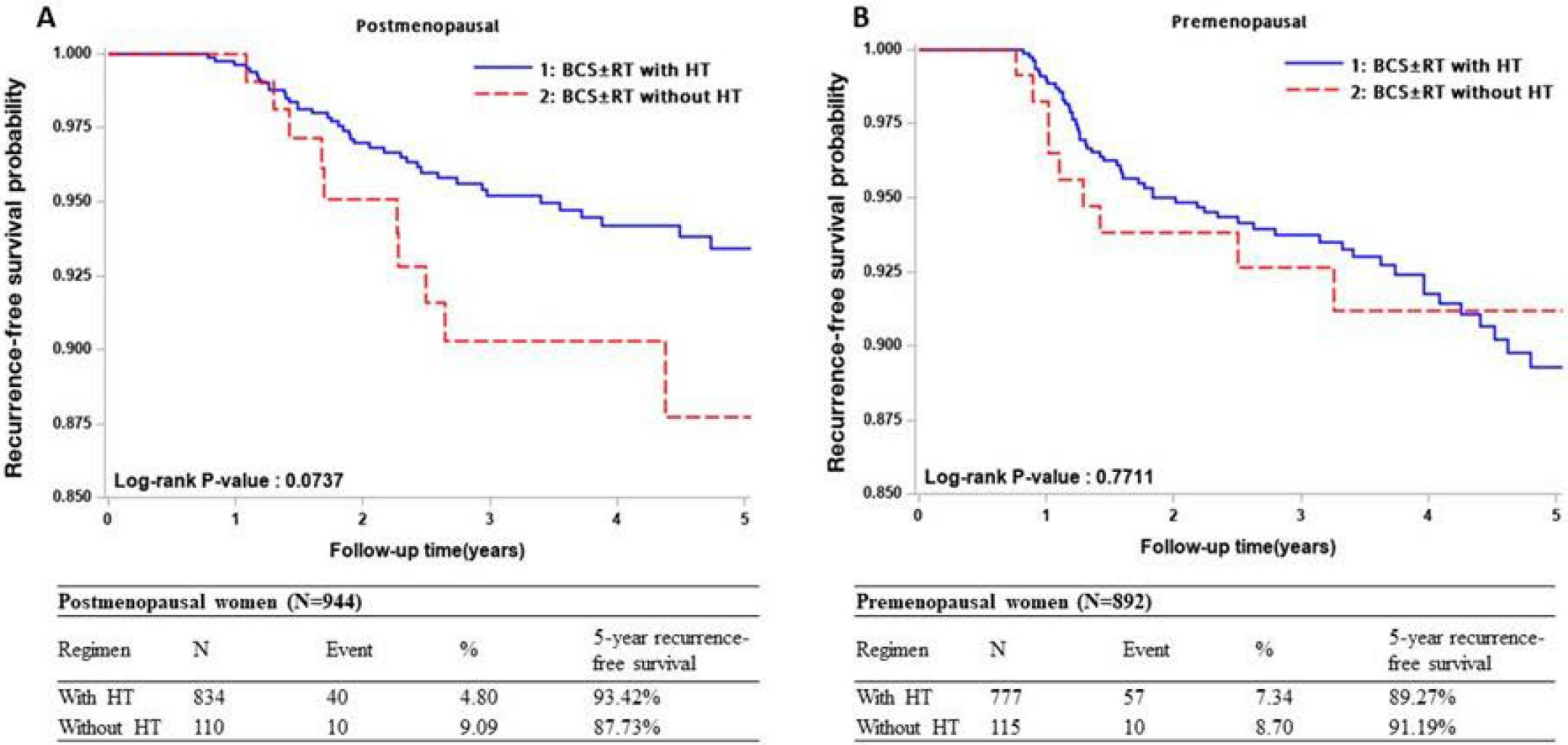
Sub-analysis: Kaplan Meier plot of cumulative recurrence-free survival between regimens with and without hormone therapy. (A) Postmenopausal, (B) Premenopausal. Abbreviations: BCS, breast-conserving surgery; HT, hormone therapy; RT, radiation therapy; RFS, recurrence-free survival.

For post-menopausal patients, 40 (4.80%) and 10 (9.09%) recurrence events were identified from the groups receiving regimens with HT (BCS±RT+HT, n = 834) and without HT (BCS±RT, n = 110), respectively (Fig 3A). In comparison, 57 (7.34%) and 10 (8.70%) recurrent events were identified from regimens with (n = 777) and without HT (n = 115), respectively in pre-menopausal patients (Fig 3B). In the post-menopausal group, the 5-year RFS was significantly higher in patients who received HT than those without HT (93.42% vs. 87.73%). On the contrary, there was no difference between these regimens in the pre-menopausal patients.

## Discussion

This population-based study investigated evidence that may inform an ongoing debate on de-escalating treatments of DCIS, particularly in patients with low-to-intermediate recurrence risk. Similar to the previous literature, this study found that the recommended treatment regimen (BCS+HT+RT) resulted in a significantly lower risk of recurrent than the regimen of BCS alone without any adjuvant therapy in patients with HR(+)-DCIS, i.e., low-to-intermediate recurrence risk. However, combining BCS with dual adjuvant therapies acquired no additional benefits than single adjuvant therapy. The recurrent risk of both BCS+RT or BCS+HT regimen was comparable to BCS+HT+RT. Moreover, the five-year RFS rate was higher in the BCS+RT group than in the BCS+HT+RT group. Compared to the addition of HT to BCS, the addition of RT might be more critical to reduce the subsequent recurrence risk. Furthermore, the dominating advantage of single over dual adjuvant therapies was not observed in patients with a high risk of recurrence (S1 Fig). Therefore, these results support the proposition to de-escalate adjuvant treatments in patients with low-to-intermediate risk of DCIS recurrence.

Previous UK/ANZ DCIS trial indicated the effectiveness of single adjuvant therapy when investigated the risk of any breast cancer event occurred in patients with locally excised DCIS comparing BCS+HT versus BCS alone (HR: 0.71, 95%CI: 0.57–0.87) or BCS+RT versus BCS alone (HR: 0.41, 95%CI: 0.30–0.57). When comparing the dual and single adjuvant therapies, this trial demonstrated that single adjuvant RT did not incur a significantly higher risk than dual therapy (BCS+RT+HT vs. BCS+RT, HR: 0.99, 95%CI: 0.61–1.59), but single adjuvant HT seems to have a higher risk of recurrence compared to dual therapy (BCS+RT+HT vs. BCS+HT, HR: 0.44, 95%CI: 0.32–0.60) [16].

In line with the consensus guideline that RT was recommended as an adjuvant method to reduce recurrence risk [33], our study found BCS+RT resulted in a significantly lower 5-year recurrence rate than BCS alone (6.14% vs. 11.71%). These results were similar to a previous study that synthesized data from randomized controlled trials and reported BCS+RT with a significantly lower 5-year ipsilateral breast event rate than BCS alone (7.6% vs. 18.1%, *P<*0.0001) in all patients with DCIS [34]. The slight differences observed may be attributed to the different study cohorts; our study included only an HR(+) low-to-intermediate risk population, previously determined to have a low recurrence risk [10]. Moreover, the results of a large matched cohort study also support the effectiveness of single adjuvant RT. Giannakeas *et al*. reported that the 15-year mortality was lower with BCS+RT than with BCS alone in patients with DCIS (1.74% vs. 2.33%) [35].

Nevertheless, the effectiveness of single adjuvant HT is less conclusive. Our study found the recurrent event rate of single adjuvant HT (BCS+HT) was significantly lower than BCS alone (7.67% vs. 11.71%) but slightly higher than BCS+RT+HT (7.67% vs. 5.42%; HR: 1.52, 95%CI: 0.99–2.35). These results echoed with the UK/ANZ DCIS trial results, and the discrepancy in effect size might be because our study only focused on the HR(+) and low-to-intermediate risk instead of all DCIS patients with BCS. In addition, the NSABP B-24 trial also reported that patients with HR(+) DCIS who received BCS+RT+tamoxifen showed a significant reduction in any breast cancer event while compared to BCS+RT (HR: 0.58, 95%CI: 0.415–0.81) [15]. The difference between the NSABP B-24 trial and our study (HR: 1.52, 95%CI: 0.99–2.35) might be because our study only included a cohort with low-to-intermediate risk.

This study adopted the scoring system for recurrence risk from Smith *et al*. (2006) using clinicopathological factors, as the information is available in the TCR database [21]. Single adjuvant RT benefited patients in either the low-to-intermediate-risk or high-risk groups. In the low-to-intermediate risk group, the absolute reduction of 5-year recurrence rate associated with RT was 9.2% (BCS+RT vs. BCS: 5.9% vs. 15.1%) (Fig 2); in the high-risk group, the corresponding reduction was 10.4% (BCS+RT vs. BCS: 15.5% vs. 25.9%) (S1 Fig). However, histological evaluation has been criticized by the high inter-observer variability and many grading/classification systems variations.

Notably, an increasing number of molecular tools, such as molecular phenotypes and genes information, are being used to de-escalate treatment components in patients with a low recurrence risk identified by expanding multi-gene expression profiling techniques. In addition to these clinicopathological factors, molecular diagnostics may offer a path to determine DCIS subtypes for de-escalating therapy in future precision medicine. For instance, Solin *et al*. created a specific panel of the Oncotype DX DCIS score (DS), calculated from seven cancer-related genes and five reference genes, that predicts LR risk after performing BCS alone on low-risk patients [36, 37]. Furthermore, Rakovitch *et al*. also demonstrated that RT reduced the 10-year LR risk in patients with a low-risk (BCS+RT vs. BCS: 5.0% vs. 10.6%) and high-risk (BCS+RT vs. BCS: 12.6% vs. 25.4%) of recurrence based on the DS score. The absolute reduction associated with RT was greater in the high-risk than low-risk group (12.8% vs. 5.6%) [38], similar to our finding.

In addition to the clinical effectiveness, adherence to treatments, adverse effects and patient preferences also influence de-escalating adjuvant therapies. A systemic review found that the prevalence of HT adherence ranged from 41 to 72% over periods greater than four years among breast cancer survivors. The discontinuation (i.e., non-persistence) rate ranged from 31 to 73% measured at the end of five years of HT treatment. Side effects were negatively associated with adherence and/or persistence [39]. The CANTO study investigated patient-reported outcomes in a French prospective clinical cohort of stage I-III breast cancer patients. Two years after the breast cancer diagnosis, 15.9% and 21.0% of patients who received adjuvant HT and RT did not return to work [40]. Moreover, in our subgroup analysis, in the postmenopausal group, treatment regimens with adjuvant HT exhibited a higher 5-year RFS than those without HT. That was not observed in the premenopausal group. One of our previous studies also resonated that older age (≥50 years) was associated with adherence to HT [41].

As the first study to focus on patients with HR(+) DCIS in a Taiwanese population, the results can inform the de-escalating therapeutic strategy in patients with low-to-intermediate risk of recurrence. To compare the single and dual adjuvant therapies in this Asian population, we selected a representative cohort with HR positivity [42], i.e., a relatively high percentage of HR(+) patients. Furthermore, this study pioneered DCIS investigation in a low-to-intermediate risk local population categorized by risk scores compared with other studies. Because the risk score was a significant predictor of breast cancer recurrence [21], selecting a similar low-intermediate risk cohort was necessary to address the effectiveness of de-escalated treatment.

We acknowledge several limitations of this study. First, for the completion of biomarker information, only patients diagnosed after 2011 were included; besides, our dataset was available 2010–2017, resulting in a shorter follow-up period. However, the reported peak for breast cancer recurrence in patients with DCIS was between the first and second years after diagnosis [43]; our study findings could reference clinicians for considering the interventions during this period. Second, we lacked confirmed ipsilateral breast tumor recurrence and margin information from our database; therefore, we used any surgery or chemotherapy records identified in the NHI database after nine months from the index date as our surrogate definition of recurrence. However, the reason for some patients receiving an indication of a second surgery may be owing to positive margins or other causes and not because of DCIS recurrence. Consequently, the biological behavior may not be so aggressive. Finally, regarding the adherence to HT, our previous study [41] indicated the proportion of non-adherence to adjuvant HT in the whole HT prescribed period was slightly lower (15.6%) in breast cancer women who have prescribed HT while compared to most of the other published observational studies range from 12% to 59% depending on the definition of each study [39].

In conclusion, in an Asian population of non-high-risk HR(+) DCIS, the combination of dual adjuvant therapies showed no significant additional benefits and was associated with unnecessary escalation. Simultaneously, the undesirable adverse effects of combined dual adjuvant therapies and patient quality of life should be considered during individual decision-making. The present study provided information to help clinicians avoid specific adjuvant treatments in patients with low-intermediate risk HR(+) DCIS. We believe that the results from the ongoing active surveillance trials, genomic prognostic testing, molecular biomarkers, and artificial intelligence tools will be helpful to provide more targeted, personalized treatment options for women with DCIS.

## Supporting information

S1 Fig Kaplan Meier plot of cumulative recurrence-free survival among BCS-based regimens in a high-risk group. Abbreviations: BCS, breast-conserving surgery; HT, hormone therapy; RT, radiation therapy.
(TIF)

## References

[1] May DS, Lee NC, Richardson LC, Giustozzi AG, Bobo JK. Mammography and breast cancer detection by race and Hispanic ethnicity: results from a national program (United States). Cancer Causes Control. 2000;11(8):697–705. doi: 10.1023/a:1008900220924 11065006

[2] Kerlikowske K, Grady D, Barclay J, Sickles EA, Eaton A, Ernster V. Positive predictive value of screening mammography by age and family history of breast cancer. JAMA,. 1993;270(20):2444–50. 8230621

[3] Ernster VL, Ballard-Barbash R, Barlow WE, Zheng Y, Weaver DL, Cutter G, et al. Detection of ductal carcinoma in situ in women undergoing screening mammography. J Natl Cancer Inst. 2002;94(20):1546–54. doi: 10.1093/jnci/94.20.1546 12381707

[4] National Comprehensive Cancer Network. Breast Cancer (Version 4, 2021) 2021 [cited 2021 14 May]. Available from: https://www.nccn.org/professionals/physician_gls/pdf/breast.pdf. 10.6004/jnccn.2021.0023 34794122

[5] Bijker N, Meijnen P, Peterse JL, Bogaerts J, Van Hoorebeeck I, Julien JP, et al. Breast-conserving treatment with or without radiotherapy in ductal carcinoma-in-situ: ten-year results of European Organisation for Research and Treatment of Cancer randomized phase III trial 10853—a study by the EORTC Breast Cancer Cooperative Group and EORTC Radiotherapy Group. J Clin Oncol. 2006;24(21):3381–7. doi: 10.1200/JCO.2006.06.1366 16801628

[6] Goodwin A, Parker S, Ghersi D, Wilcken N. Post-operative radiotherapy for ductal carcinoma in situ of the breast. Cochrane Database Syst Rev. 2009(4):Cd000563. 10.1002/14651858.CD000563.pub5 19588320

[7] Holmberg L, Garmo H, Granstrand B, Ringberg A, Arnesson LG, Sandelin K, et al. Absolute risk reductions for local recurrence after postoperative radiotherapy after sector resection for ductal carcinoma in situ of the breast. J Clin Oncol. 2008;26(8):1247–52. doi: 10.1200/JCO.2007.12.7969 18250350

[8] Correa C, McGale P, Taylor C, Wang Y, Clarke M, Davies C, et al. Overview of the randomized trials of radiotherapy in ductal carcinoma in situ of the breast. J Natl Cancer Inst Monogr. 2010;2010(41):162–77. doi: 10.1093/jncimonographs/lgq039 20956824 PMC5161078

[9] Choi YJ, Shin YD, Song YJ. Comparison of ipsilateral breast tumor recurrence after breast-conserving surgery between ductal carcinoma in situ and invasive breast cancer. World J Surg Oncol. 2016;14:126. doi: 10.1186/s12957-016-0885-6 27122132 PMC4848787

[10] Williams KE, Barnes NLP, Cramer A, Johnson R, Cheema K, Morris J, et al. Molecular phenotypes of DCIS predict overall and invasive recurrence. Ann Oncol. 2015;26(5):1019–25. doi: 10.1093/annonc/mdv062 25678586

[11] Lazzeroni M, Dunn BK, Pruneri G, Jereczek-Fossa BA, Orecchia R, Bonanni B, et al. Adjuvant therapy in patients with ductal carcinoma in situ of the breast: The Pandora’s box. Cancer Treat Rev. 2017;55:1–9. doi: 10.1016/j.ctrv.2017.01.010 28262606

[12] Grantzau T, Mellemkjaer L, Overgaard J. Second primary cancers after adjuvant radiotherapy in early breast cancer patients: a national population based study under the Danish Breast Cancer Cooperative Group (DBCG). Radiother Oncol. 2013;106(1):42–9. doi: 10.1016/j.radonc.2013.01.002 23395067

[13] Henson KE, McGale P, Taylor C, Darby SC. Radiation-related mortality from heart disease and lung cancer more than 20 years after radiotherapy for breast cancer. Br J Cancer. 2013;108(1):179–82. doi: 10.1038/bjc.2012.575 23257897 PMC3553540

[14] Ganz PA, Cecchini RS, Julian TB, Margolese RG, Costantino JP, Vallow LA, et al. Patient-reported outcomes with anastrozole versus tamoxifen for postmenopausal patients with ductal carcinoma in situ treated with lumpectomy plus radiotherapy (NSABP B-35): a randomised, double-blind, phase 3 clinical trial. Lancet. 2016;387(10021):857–65. doi: 10.1016/S0140-6736(15)01169-1 26686960 PMC4792658

[15] Allred DC, Anderson SJ, Paik S, Wickerham DL, Nagtegaal ID, Swain SM, et al. Adjuvant tamoxifen reduces subsequent breast cancer in women with estrogen receptor-positive ductal carcinoma in situ: a study based on NSABP protocol B-24. J Clin Oncol. 2012;30(12):1268–73. doi: 10.1200/JCO.2010.34.0141 22393101 PMC3341142

[16] Cuzick J, Sestak I, Pinder SE, Ellis IO, Forsyth S, Bundred NJ, et al. Effect of tamoxifen and radiotherapy in women with locally excised ductal carcinoma in situ: long-term results from the UK/ANZ DCIS trial. Lancet Oncol. 2011;12(1):21–9. doi: 10.1016/S1470-2045(10)70266-7 21145284 PMC3018565

[17] Forbes JF, Sestak I, Howell A, Bonanni B, Bundred N, Levy C, et al. Anastrozole versus tamoxifen for the prevention of locoregional and contralateral breast cancer in postmenopausal women with locally excised ductal carcinoma in situ (IBIS-II DCIS): a double-blind, randomised controlled trial. Lancet. 2016;387(10021):866–73. doi: 10.1016/S0140-6736(15)01129-0 26686313 PMC4769326

[18] Margolese RG, Cecchini RS, Julian TB, Ganz PA, Costantino JP, Vallow LA, et al. Anastrozole versus tamoxifen in postmenopausal women with ductal carcinoma in situ undergoing lumpectomy plus radiotherapy (NSABP B-35): a randomised, double-blind, phase 3 clinical trial. Lancet. 2016;387(10021):849–56. doi: 10.1016/S0140-6736(15)01168-X 26686957 PMC4792688

[19] Wapnir IL, Dignam JJ, Fisher B, Mamounas EP, Anderson SJ, Julian TB, et al. Long-term outcomes of invasive ipsilateral breast tumor recurrences after lumpectomy in NSABP B-17 and B-24 randomized clinical trials for DCIS. J Natl Cancer Inst. 2011;103(6):478–88. doi: 10.1093/jnci/djr027 21398619 PMC3107729

[20] Silverstein MJ. The University of Southern California/Van Nuys prognostic index for ductal carcinoma in situ of the breast. Am J Surg. 2003;186(4):337–43. doi: 10.1016/s0002-9610(03)00265-4 14553846

[21] Smith GL, Smith BD, Haffty BG. Rationalization and regionalization of treatment for ductal carcinoma in situ of the breast. Int J Radiat Oncol Biol Phys. 2006;65(5):1397–403. doi: 10.1016/j.ijrobp.2006.03.009 16750316

[22] Nofech-Mozes S, Hanna W, Rakovitch E. Molecular Evaluation of Breast Ductal Carcinoma in Situ with Oncotype DX DCIS. Am J Pathol. 2018;189(5):975–80. doi: 10.1016/j.ajpath.2018.12.003 30605628

[23] Chiang JK, Lin CW, Wang CL, Koo M, Kao YH. Cancer studies based on secondary data analysis of the Taiwan’s National Health Insurance Research Database: A computational text analysis and visualization study. Medicine (Baltimore). 2017;96(17):e6704. doi: 10.1097/MD.0000000000006704 28445277 PMC5413242

[24] Health Promotion Administration. Taiwan Breast Cancer Screening Program [in Chinese] 2015 [cited 2021 14 May]. Available from: https://www.hpa.gov.tw/Pages/Detail.aspx?nodeid=1129&pid=2045.

[25] Narod SA, Iqbal J, Giannakeas V, Sopik V, Sun P. Breast Cancer Mortality After a Diagnosis of Ductal Carcinoma In Situ. JAMA Oncol. 2015;1(7):888–96. doi: 10.1001/jamaoncol.2015.2510 26291673

[26] Shamliyan T, Wang SY, Virnig BA, Tuttle TM, Kane RL. Association between patient and tumor characteristics with clinical outcomes in women with ductal carcinoma in situ. J Natl Cancer Inst Monogr. 2010;2010(41):121–9. doi: 10.1093/jncimonographs/lgq034 20956815 PMC5161074

[27] Tunon-de-Lara C, Andre G, Macgrogan G, Dilhuydy JM, Bussieres JE, Debled M, et al. Ductal carcinoma in situ of the breast: influence of age on diagnostic, therapeutic, and prognostic features. Retrospective study of 812 patients. Ann Surg Oncol. 2011;18(5):1372–9. doi: 10.1245/s10434-010-1441-1 21108045

[28] Pistelli M, Mora AD, Ballatore Z, Berardi R. Aromatase inhibitors in premenopausal women with breast cancer: the state of the art and future prospects. Curr Oncol. 2018;25(2):e168–e75. doi: 10.3747/co.25.3735 29719441 PMC5927796

[29] Chen SC, Lo TC, Chang JH, Kuo HW. Variations in aging, gender, menopause, and obesity and their effects on hypertension in taiwan. Int J Hypertens. 2014;2014:515297. doi: 10.1155/2014/515297 25436143 PMC4243128

[30] Kong I, Narod SA, Taylor C, Paszat L, Saskin R, Nofech-Moses S, et al. Age at diagnosis predicts local recurrence in women treated with breast-conserving surgery and postoperative radiation therapy for ductal carcinoma in situ: a population-based outcomes analysis. Curr Oncol. 2013;21(1):e96–e104. 10.3747/co.21.1604 PMC3921054 24523627

[31] Sue GR, Lannin DR, Killelea B, Tsangaris T, Chagpar AB. Does time to definitive treatment matter in patients with ductal carcinoma in situ? Am Surg. 2013;79(6):561–5. 23711263

[32] Charlson ME, Pompei P, Ales KL, MacKenzie CR. A new method of classifying prognostic comorbidity in longitudinal studies: development and validation. J Chronic Dis. 1987;40(5):373–83. doi: 10.1016/0021-9681(87)90171-8 3558716

[33] National Comprehensive Cancer Network. Breast Cancer (Version 4, 2018) 2019 [cited 2021 14 May]. Available from: https://www.nccn.org/professionals/physician_gls/pdf/breast.pdf.

[34] Early Breast Cancer Trialists’ Collaborative Group, Correa C, McGale P, Taylor C, Wang Y, Clarke M, et al. Overview of the randomized trials of radiotherapy in ductal carcinoma in situ of the breast. J Natl Cancer Inst Monogr. 2010;2010(41):162–77. doi: 10.1093/jncimonographs/lgq039 20956824 PMC5161078

[35] Giannakeas V, Sopik V, Narod SA. Association of Radiotherapy With Survival in Women Treated for Ductal Carcinoma In Situ With Lumpectomy or Mastectomy. JAMA Netw Open. 2018;1(4):e181100. doi: 10.1001/jamanetworkopen.2018.1100 30646103 PMC6324271

[36] Rakovitch E, Nofech-Mozes S, Hanna W, Baehner FL, Saskin R, Butler SM, et al. A population-based validation study of the DCIS Score predicting recurrence risk in individuals treated by breast-conserving surgery alone. Breast Cancer Res Treat. 2015;152(2):389–98. doi: 10.1007/s10549-015-3464-6 26119102 PMC4491104

[37] Solin LJ, Gray R, Baehner FL, Butler SM, Hughes LL, Yoshizawa C, et al. A multigene expression assay to predict local recurrence risk for ductal carcinoma in situ of the breast. J Natl Cancer Inst. 2013;105(10):701–10. doi: 10.1093/jnci/djt067 23641039 PMC3653823

[38] Rakovitch E, Nofech-Mozes S, Hanna W, Sutradhar R, Baehner FL, Miller DP, et al. Multigene Expression Assay and Benefit of Radiotherapy After Breast Conservation in Ductal Carcinoma in Situ. J Natl Cancer Inst. 2017;109(4):djw256. doi: 10.1093/jnci/djw256 30053207 PMC6233855

[39] Murphy CC, Bartholomew LK, Carpentier MY, Bluethmann SM, Vernon SW. Adherence to adjuvant hormonal therapy among breast cancer survivors in clinical practice: a systematic review. Breast Cancer Res Treat. 2012;134(2):459–78. doi: 10.1007/s10549-012-2114-5 22689091 PMC3607286

[40] Dumas A, Vaz Luis I, Bovagnet T, El Mouhebb M, Di Meglio A, Pinto S, et al. Impact of Breast Cancer Treatment on Employment: Results of a Multicenter Prospective Cohort Study (CANTO). J Clin Oncol. 2020;38(7):734–43. doi: 10.1200/JCO.19.01726 31834818 PMC7048162

[41] Hsieh KP, Chen LC, Cheung KL, Yang YH. Risks of nonadherence to hormone therapy in Asian women with breast cancer. Kaohsiung J Med Sci. 2015;31(6):328–34. doi: 10.1016/j.kjms.2015.04.002 26043413 PMC11916748

[42] Bailes AA, Kuerer HM, Lari SA, Jones LA, Brewster AM. Impact of race and ethnicity on features and outcome of ductal carcinoma in situ of the breast. Cancer. 2013;119(1):150–7. doi: 10.1002/cncr.27707 22736444 PMC3461121

[43] Colleoni M, Sun Z, Price KN, Karlsson P, Forbes JF, Thurlimann B, et al. Annual Hazard Rates of Recurrence for Breast Cancer During 24 Years of Follow-Up: Results From the International Breast Cancer Study Group Trials I to V. J Clin Oncol. 2016;34(9):927–35. doi: 10.1200/JCO.2015.62.3504 26786933 PMC4933127

