# Supplementary figures and images for: Investigating the effectiveness of adjuvant therapy for patients with hormone receptor-positive ductal carcinoma in situ
Source: PLoS One. 2022 Jan 28;17(1):e0262934. doi: 10.1371/journal.pone.0262934 (PMC8797251; doi:10.1371/journal.pone.0262934)

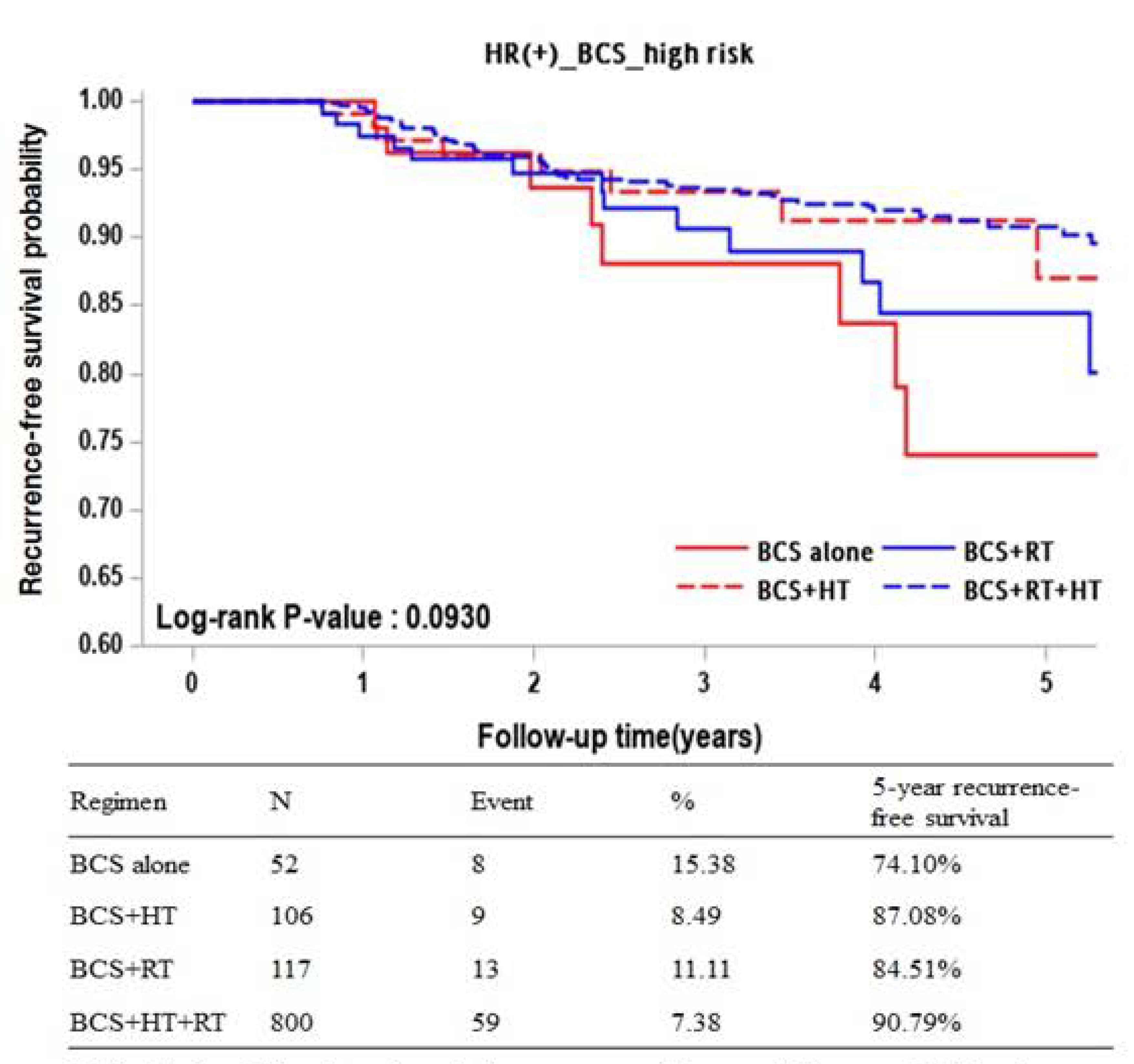

Supplement: S1 Fig — Abbreviations: BCS, breast-conserving surgery; HT, hormone therapy; RT, radiation therapy. (TIF) [file pone.0262934.s001.tif]
